# Supplementary material for: DMRforPairs: identifying Differentially Methylated Regions between unique samples using array based methylation profiles
Source: BMC Bioinformatics. 2014 May 15;15:141. doi: 10.1186/1471-2105-15-141 (PMC4046028; doi:10.1186/1471-2105-15-141)
Supplement: Additional file 2 — DMRforPairs output for the comparison of A431-MCF7 and NA17018-NA17105. Please start from the HTML files in each folder. Available via the BMC Bioinformatics website. [file 1471-2105-15-141-S2.zip › 1394847754114233_MOESM2_ESM/NA17105M_NA17018F/figures/251.pdf]

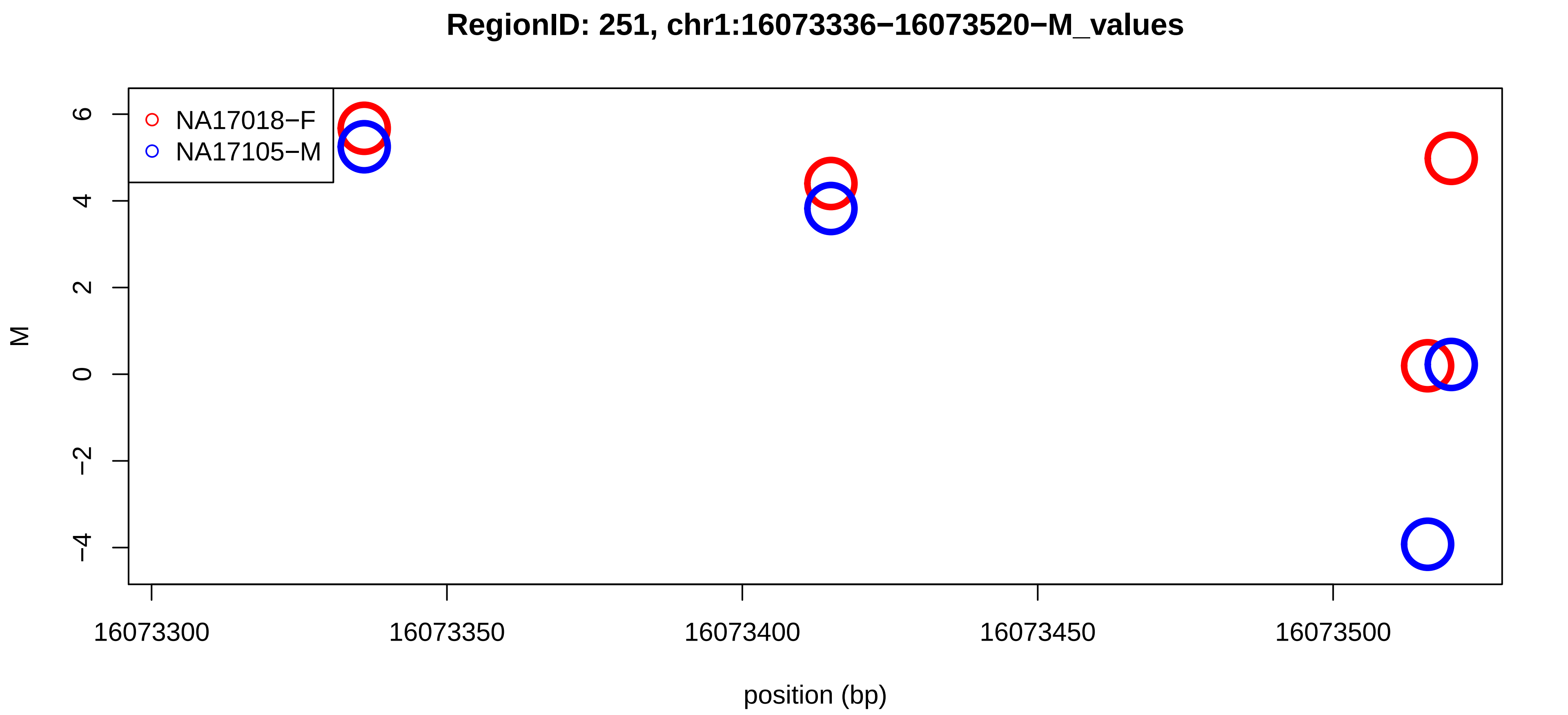
RegionID: 251, chr1:16073336−16073520−M_values
NA17018−F
NA17105−M
M
6
4
2
0
−2
−4
16073300
16073350
16073400
16073450
16073500
position (bp)

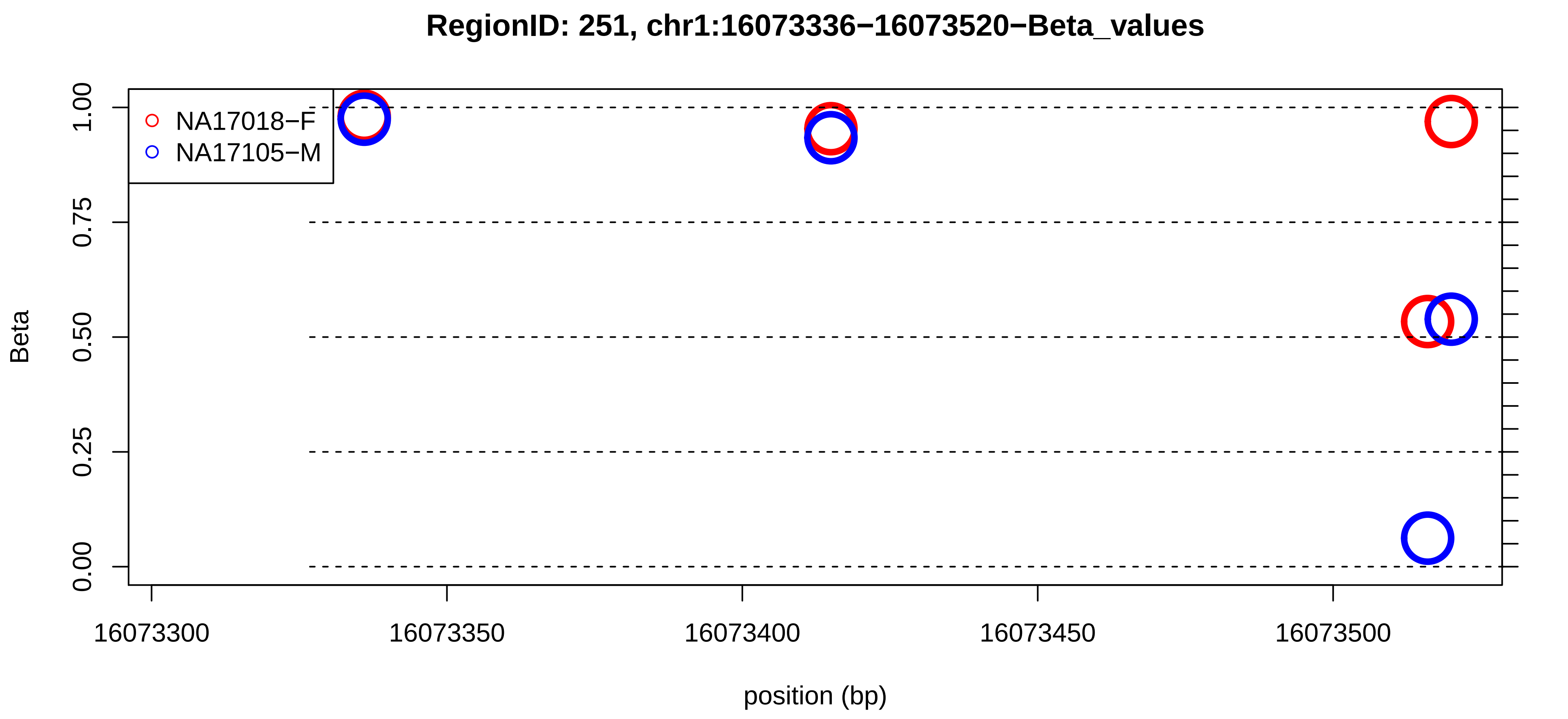
RegionID: 251, chr1:16073336−16073520−Beta_values
NA17018−F
NA17105−M
Beta
1.00
0.75
0.50
0.25
0.00
16073300
16073350
16073400
16073450
16073500
position (bp)
